# The potential role of interleukin (IL)‐25/IL‐33/thymic stromal lymphopoietin (TSLP) on the pathogenesis of idiopathic pulmonary fibrosis

**DOI:** 10.1111/crj.13541

**Published:** 2022-09-09

**Authors:** Xuefeng Xu, Huaping Dai, Jinglan Zhang

**Affiliations:** ^1^ Department of Surgical Intensive Care Unit, Beijing An Zhen Hospital Capital Medical University Beijing China; ^2^ Department of Pulmonary and Critical Care Medicine, Center for Respiratory Diseases, China‐Japan Friendship Hospital National Clinical Research Center for Respiratory Diseases Beijing China

**Keywords:** epithelial–mesenchymal crosstalk, IL‐25, IL‐33, IPF, TSLP

## Abstract

**Objectives:**

Interleukin (IL)‐25, IL‐33, and thymic stromal lymphopoietin (TSLP) are the important drivers for excessive type‐2 immunity. It has been well elucidated that IL‐25/IL‐33/TSLP plays an important role in allergic airway inflammation and remodeling, whereas their roles in idiopathic pulmonary fibrosis (IPF) still remained largely unclear. Herein, the aim of the review is to discuss the potential role and mechanism of IL‐25/IL‐33/TSLP on IPF by literature analysis and summary.

**Data source:**

We have done a literature search using the following terms: (“idiopathic pulmonary fibrosis” OR “IPF” OR “lung fibrosis”) and (TSLP or “thymic stromal lymphopoietin” or IL‐25 OR IL‐17E OR IL‐33) from the database of PubMed published in English up to July 2018.

**Study selection:**

We have totally found 58 articles by using the retrieval terms mentioned above. By careful title and abstract reading, 10 original research articles of high quality were enrolled for the full text reading and analysis. Two additional relevant studies were also included during the course of literature readings.

**Results:**

IL‐25/IL‐33/TSLP and their corresponding receptors, that is, IL‐17BR/ST2L/TSLPR, are shown to be up‐regulated both in IPF patients and bleomycin (BLM)‐induced lung fibrosis mice model. IL‐25 may promote lung fibrosis by activating IL‐17BR+fibroblast and IL‐17BR+ILC2 (type 2 innate lymphoid cell). Full length (fl)‐IL‐33, as a transcription factor mainly in the cell nucleus, mediated non‐atopic lung inflammation and fibrosis by modulating expressions of several pro‐fibrotic mediators, including transforming growth factor (TGF)‐b1. By contrast, mature (m)‐IL‐33 potentiates lung fibrosis by recruiting ST2L+M2 macrophages and ST2L+ILC2 to enlarge type 2 immunity. TSLP was shown to directly promote CCL2 expression in primary human lung fibroblasts (pHLFs).

**Conclusion:**

IL‐25/IL‐33/TSLP contributes to non‐allergic lung fibrosis by mediating persistent abnormal epithelial‐mesenchymal crosstalk. IL‐25/IL‐33/TSLP may serve the promising novel target for the treatment of IPF.

## Graphical abstract

Interleukin (IL)‐25/IL‐33/thymic stromal lymphopoietin (TSLP) derived from alveolar epithelial cells (AECs) can initiate an abnormal, profibrotic crosstalk to mesenchymal cells (including fibroblasts, dendritic cell [DCs], M2‐macrophage, and type 2 innate lymphoid cells [ILC2s]) and thereby leads to lung fibrosis.

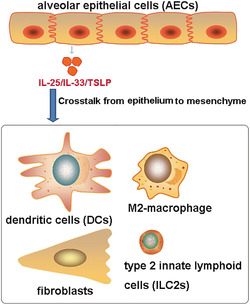

## 1 INTRODUCTION

Idiopathic pulmonary fibrosis (IPF) is a chronic, progressive, irreversible, and devastating fibrotic lung disease with unknown etiology.^1^ The worldwide incidence of IPF varies from three to nine cases per 100 000 person‐years, with an increasing rate of prevalence, hospital admissions, and deaths in more recent years.^1^ We designed one single‐center retrospective study in China and showed that 692 (26.5%) of 2615 patients with interstitial lung diseases (ILD_S_) were diagnosed with IPF, suggesting that IPF is not a rare disease, but the most common subtype of ILD_S._ ^2^ The prognosis for IPF patients is quite poor, with a median survival of 3–5 years if untreated.^3^ In this review, we will discuss the current knowledge about the pathogenesis of IPF. First, we give a brief overview of the pathogenesis and we then review in detail the potential modulatory role of interleukin (IL)‐25/IL‐33/thymic stromal lymphopoietin (TSLP) on the initiation and development of this devastating disease.

## 2 PATHOGENESIS

Historically, chronic alveolar inflammation was thought to be the key mechanism driving fibrotic lesions in IPF patients.^4^ However, pathologic studies revealed that inflammatory cells and responses are scarce. Instead, the prevailing histological features are the structures formed by injured epithelial cells and the adjacent mesenchymal compartments termed as “fibroblastic foci.”^4^ Consequently, the pathogenesis of IPF is now commonly believed as chronic/recurrent microinjuries of alveolar epithelial cells‐initiated abnormal (myo)fibroblasts activation.^4^ Specifically, injured alveolar epithelial cells produce a variety of cytokines, chemokines, and growth factors that act directly on the resident (myo)fibroblasts, which ultimately leads to the excessive collagen deposition and destruction of lung parenchyma.^4^ Therefore, this abnormal interaction between dys‐regulated alveolar epithelium and the adjacent mesenchymal cellular milieu leading to persistent activation of abnormal wound‐healing process is now considered to be the modern conceptual framework for the mechanisms of disease.^5^ However, the exact and master mediators are still not fully understood, leaving IPF still being yet an untreatable fibrotic disease despite the “Posttreatment Era” of pirfenidone and nintedanib.^6^

Studies using cytokine‐depletion/over‐expression mice showed that lung fibrosis is strongly associated with the development of a type 2 cytokine immunity, including IL‐4, IL‐5, and IL‐13,^7^, ^8^, ^9^ indicating a skew in favor of type 2 cytokine milieu may be the key molecular event mediating abnormal epithelial–mesenchymal crosstalk. However, the initiating and maintaining mechanism of type 2 polarization are remained largely unknown. Recently, activated epithelial cell derived IL‐25, IL‐33, and TSLP are reported to drive Th2‐type adaptive immune responses by binding with their corresponding receptors on various immune cells, including group 2 innate lymphoid cells (ILC2s), M2‐macrophages, dendritic cells (DCs), eosnophils, and fibroblasts.^10^, ^11^ Both human and experimental studies show that IL‐25/IL‐33/TSLP was up‐regulated in allergic airway diseases^12^, ^13^, ^14^, ^15^, ^16^, ^17^ and contributes to allergic airway inflammation and remodeling.^16^, ^18^, ^19^ However, the proinflammatory and profibrogenic role of IL‐25/IL‐33/TSLP in chronic, non‐allergic lung parenchyma remodeling is not fully elucidated, especially in IPF. Herein, this review would try to summarize and discuss the potential roles of IL‐25/IL‐33/TSLP in IPF by literature analysis (Table 1).

**TABLE 1:** Clinical and experimental studies defining the roles of IL‐25/IL‐33/TSLP on IPF

Ref.	Authors	Study design	Major findings	Conclusions
IL‐25				
^22^	Hams et al.	a: small cohort of IPF patients (*N* = 14); b: *Schistosoma mansoni* egg and BLM induced pulmonary fibrosis model.	a: increased BALF expression of IL‐25 and ILC2 in IPF patients; b: IL‐25^−/−^or IL‐17BR^−/−^ attenuated local expression of IL‐4 and IL‐13 and reduced pulmonary collagen deposition in the two fibrosis model; c: increased numbers of IL‐13^+^ILC2 following egg challenge; d: ILC2‐depletion dampened pulmonary collagen deposition, reduced IL‐13 and TGFβ1 expression induced by IL‐25 and egg; e: transfer of IL‐13^+^ILC2 significantly induced collagen deposition.	IL‐25 promotes lung fibrosis in a IL‐13^+^IL‐17BR^+^ILC2 dependent fashion.
^24^	Létuvé et al.	In vitro cell culture of human primary lung fibroblasts.	a: IL‐17BR was constitutively expressed by human primary lung fibroblasts; b: IL‐25 increased expression levels of CCL–5, CCL‐11, GM‐CSF and CXCL‐8; c: TNF‐α potentiated IL‐25's role on induction of GM‐CSF and CXCL‐8.	IL‐25 potentiates the expressions of pro‐inflammatory cytokines by directly acting on lung fibroblasts.
^25^	Lee et al.	Large cohort of IPF patients (*N* = 100).	BALF expression level of IL‐25 were not significantly different between Normal control and IPF group	Alveolar IL‐25 was not up‐regulated in IPF.
IL‐33				
^27^	Tajima et al.	Retrospective study with 49 IPF patients.	a: the serum levels of sST2 in AE‐IPF patients were significantly higher than those in IPF patients with stable state and healthy subjects; b: serum ST2 correlated positively with LDH and CRP, and negatively correlated with PaO2 and VC%.	sST2 protein may be the potential biomarker for the inflammatory process in the IPF lung.
^28^	Tajima et al.	a: BLM‐induced lung fibrosis model; b: in vitro cell culture of human primary lung fibroblasts and AECs.	a: ST2 gene was markedly induced following with the upregulation of Th2‐type cytokines (IL‐4 and IL‐5) and proinflammatory cytokines (IL‐1β and TNF‐α) induced by BLM; b: IL‐1β, TNF‐α and IL‐4 could promote ST2 mRNA expression in both AECs and lung fibroblast.	Elevated sT2 level reflects dysregulated local inflammatory response and the Th2‐type immune milieu in lung fibrosis.
^29^	Luzina et al.	a: small cohort with 3 patients with IPF; b: BLM‐induced lung fibrosis model.	a: hIL‐33 constitutively expressed in healthy lungs; b: hIL‐33 was much higher in the lungs of IPF patients than control subjects, with inflammatory cells and fibroblasts to be the most intense staining cells; c: flmIL‐33 recruited lymphocytes and neutrophils, and promoted the production of IL‐1β, TNF‐α, IL‐6, MIP‐1a and RANTES; d: ST2L deficiency failed to attenuate flmIL‐33 mediated pulmonary infiltration and the production of the proinflammatory cytokines.	flmIL‐33 mediates non‐atopic lung inflammation in a ST2L‐independent manner
^30^	Luzina et al.	a: small cohort with 11 patients with IPF; b: BLM‐induced lung fibrosis model.	a: infiltrating cells and stromal cells in IPF lung showed obvious positive staining of IL‐33; b: flmIL‐33 transfection induced significant lung collagen deposition; c: flmIL‐33 transfection further potentiated lung collagen deposition induced by BLM; d: flmIL‐33 plus BLM synergistically aggregated the expression of TGF‐β, MCP‐1, MIP‐1α, IL‐6, TNF‐α, and HSP70	flIL‐33 exhibits a proinflammatory and profibrotic effect in a ST2L and Th2‐independent fashion.
^31^	Li et al.	BLM‐induced lung fibrosis model.	a: IL‐33 is constitutively expressed in AECs but can be induced in macrophages by BLM; b: ST2 depletion/anti–IL‐33 antibody/alveolar macrophage depletion attenuated bleomycin‐induced IL‐33, IL‐13, TGF‐β1 expression, and disrupted lung collagen deposition. c: Exogenous IL‐33/transfer of ILC2s further potentiated lung inflammation and fibrosis induced by BLM; d: IL‐33 polarized M2 macrophages to produce IL‐13 and TGF‐β1 and induced the expansion of ILC2s to produce IL‐13.	mIL‐33‐ST2L axis potentiates lung inflammation and fibrosis in a M2 macrophages and ILC2s dependent manner.
^33^	Gao et al.	BLM‐induced lung fibrosis model.	a: sST2 treatment significantly improved survival rate and reduced weight loss induced by BLM challenge; b: sST2 treatment markedly lowered the BALF levels of IL‐4, IL‐6, IL‐13, IL‐33, MCP‐1, TGF‐β1, whereas it increased the levels of IFN‐γ; c: sST2 treatment profoundly attenuated the pulmonary inflammatory cell infiltration and fibrotic changes.	sST2 attenuated lung fibrosis by blocking IL‐33/STL2 axis and down‐regulating proinflammatory and profibrotic mediators.
^34^	Fanny et al.	BLM‐induced lung fibrosis model.	a: 24 h post‐BLM treatment ST2‐deficient mice displayed augmented inflammatory cell recruitment and edema; b: lung remodeling and fibrosis were decreased with reduced M2 macrophages associated with M2‐like cytokine profile in ST2‐deficient mice.	IL‐33/ST2 promotes lung fibrosis by potentiating M2 macrophages skew.
^35^	Zhao et al.	a: BLM‐induced lung fibrosis model; b: in vitro cell culture of ILC2/fibroblast	a: ILC2 recruitment, IL‐13 induction, and fibrosis were significantly diminished in ST2‐deficient‐BM chimera mice; b: ILC2 from bleomycin‐treated mice stimulated type I collagen expression in lung fibroblast.	IL‐33/ST2 pathway contributed to fibroblast activation to promote lung fibrosis by recruiting BM‐derived ILC2s.
^25^	Lee et al.	Large cohort of IPF patients (*N* = 100).	BALF expression level of IL‐33 were significantly up‐regulated in patients with IPF than normal controls	IL‐33 mediated innate immune responses may be associated with the development of IPF.
TSLP				
^40^	Datta et al.	a: small cohort of IPF patients (*N* = 12) b: in vitro cell culture of fibroblast	a: TSLP were up‐regulated in AECs, myofibroblasts in IPF lung; b: TSLPR immunostaining was positive on ASMCs, AECs and myofibroblasts in IPF lung; c: TSLP increased CCL2 expression by pHLFs	TSLP promotes lung fibrosis by directly acting on lung fibroblast.
^25^	Lee et al.	Large cohort of IPF patients (*N* = 100).	BALF expression level of TSLP were significantly up‐regulated in patients with IPF than normal controls	TSLP mediated innate immune responses may be associated with the development of IPF.

Abbreviations: AECs, alveolar epithelial cells; ASMCs, airway smooth muscle cells; BALF, bronchoalveolar lavage fluid; BLM, bleomycin; BM, bone marrow; CRP, C‐reactive protein; HSP70, heat shock protein 70; IFN‐γ, interferon‐γ; ILC2s, type 2 innate lymphoid cells; IPF, idiopathic pulmonary fibrosis; LDH, lactate dehydrogenase; MCP‐1, monocyte chemoattractant protein‐1; MIP‐1α, macrophage inflammatory protein 1α; pHLFs, PaO_2_, arterial partial pressure of oxygen; pulmonary human lung fibroblasts; sST2, soluble ST2; TGF‐β1, transforming growth factor‐β1; TNF‐α, tumor necrosis factor α; TSLP, thymic stromal lymphopoietin; VC%, vital capacity%.

## 3 IL‐25/IL‐17BR AXIS IN THE PATHOGENESIS OF IPF: DEPENDING ON ILC2S AND FIBROBLAST

As has been described before, it has been well elucidated that IL‐25/IL‐17BR plays a significant role in allergic airway diseases, especially in allergic asthma.^18^, ^20^, ^21^ However, whether IL‐25/IL‐17BR axis is involved in the pathogenesis of non‐atopic lung inflammation and fibrosis (especially IPF) is still not fully explored. We found only one important study that explored the potential role of IL‐25 in both murine models and human IPF conditions. In their study, Hams et al.^22^ first reported that deficiency in IL‐25 (i.e., IL‐25^−/−^) and its receptor IL‐17BR (i.e., IL‐17BR^−/−^) obviously attenuated local expression of type 2 cytokines (including IL‐4 and IL‐13) and reduced pulmonary collagen deposition in both *Schistosoma mansoni* egg and bleomycin (BLM)‐induced lung fibrosis model. They also found that there was a significant increase in the numbers of lung and mediastinal lymph node (MLN) IL‐13^+^‐ILC2 after *S. mansoni* egg challenge, which was notably reduced by depletion of IL‐25 and IL‐17BR. ILC2s are as subset of innate immune cells, which have been shown to involve in abnormal tissue repair responses through the excessive production of amphiregulin and IL‐13.^23^ In accordance with this study, Hams et al.^22^ showed that ILC2‐depletion remarkably inhibited pulmonary collagen deposition and residential IL‐13/transforming growth factor (TGF)‐β1 expression induced by *S. mansoni* egg or IL‐25 instillation, indicating IL‐13^+^‐ILC2 mediated IL‐25/IL‐17BR's role on the development of pulmonary fibrosis. This was further confirmed by their following experiments which showed that transfer of IL‐13^+^ILC2 significantly potentiated collagen deposition, and further amplified lung fibrosis induced by *S. mansoni egg*.

More importantly, by using bronchoalveolar lavage fluid (BALF) from a small cohort of IPF patients (*N* = 14), the authors^22^ showed that the expression level of IL‐25 was significantly up‐regulated at initial diagnosis and further induced 1 year after diagnosis. BALF cellularity analysis also demonstrated that a population of (lineage^−^ ICOS^+^ CD45^+^ CRTH2^+^ T1/ST2^+^ IL‐17BR^+^)‐ILC2 was significantly increased when compared with control patients, which was combined with the elevated BLAF expressions of several pro‐inflammatory and pro‐fibrogenic cytokines, including IL‐1β, IL‐2, IL‐4, IL‐5, IL‐8, IL‐12p70, IL‐13, IL‐17A, interferon (IFN)‐γ, and tumor necrosis factor (TNF)‐α. Taken together, this study highlights that IL‐25/IL‐17BR axis may underlie the pathogenesis of IPF in a (IL‐13^+^IL‐17BR^+^)‐ILC2 dependent manner.

**FIGURE 1:**
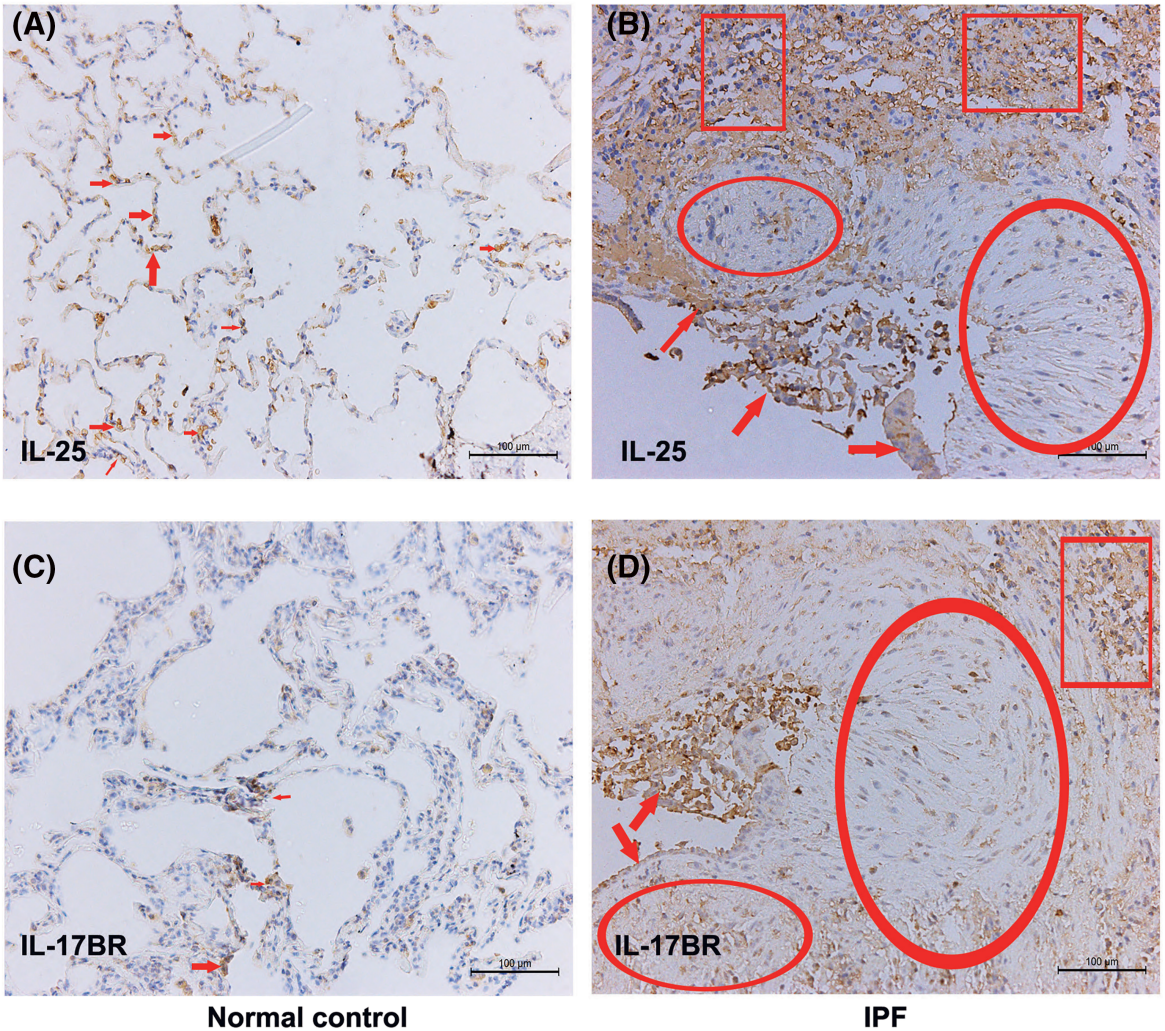
Expression levels of IL‐25/IL‐17BR in IPF lungs. Shown is immunohistochemical staining for IL‐25 (A and B) and IL‐17BR (C and D) in lung sections of IPF patients and normal controls. (A) In normal lung, positive staining for IL‐25 is observed in alveolar epithelial cells (red arrows). (B) In IPF lung, strong immuno‐reactivity for IL‐25 is showed in shedding alveolar epithelial cells (red arrows) and less intensively in fibroblasts within fibrotic foci (red scoops). Immuno‐staining is also obviously associated with infiltrated lymphocytes (red squares). (C) In normal lung, positive staining for IL‐17BR is occasionally observed in alveolar epithelial cells (red arrows). (D) In IFP lung, IL‐17BR staining is also observed in alveolar epithelial cells, fibroblasts and infiltrated lymphocytes as IL‐25. Scale bars indicate 100 μm. Abbreviations: IL, interleukin; IPF, idiopathic pulmonary fibrosis

In line with this study, by using immunohistochemistry staining, we showed both IL‐25 and IL‐17BR were up‐regulated in lung tissues of IPF patients (Figure 1). IL‐25 and IL‐17BR‐expressing cells were abundant in the type II alveolar epithelial cells. Although less intensely stained, mesenchymal cells resided in the fibrotic foci (mainly myofibroblasts) also clearly express IL‐25 and IL‐17BR. This indicates that in addition to ILC2, IL‐25 may exert its profibrotic effect by mediating epithelial cell injury and myofibroblast activation in a paracrine or autocrine fashion. This was further confirmed by Létuvé et al.'s study, which demonstrated that IL‐17BR is highly expressed in cultured primary human lung fibroblasts and that IL‐25 administration potentiated the production of the eosinophil‐associated cytokines, including CCL‐5, CCL‐11, granulocyte‐macrophage colony stimulating factor (GM‐CSF), and CXCL‐8.^24^

Collectively, we suggest that IL‐25/IL‐17BR axis is highly activated in patients with IPF, which exerts pro‐fibrotic effect via acting on IL‐17BR^+^‐ILC2 and ‐myofibroblast. However, Lee et al.^25^ recruited a relatively large cohort of patients with IPF (*N* = 100) to show that the expression level of alveolar IL‐25 (IL‐25 in BALF) was comparable between IPF patients and the normal controls. The exact reason for the discrepancy on the expression levels of alveolar IL‐25 in IPF patients is remained unclear. However, different phenotypes of enrolled patients, different BALF collection methods, and detection methods may serve as the possible interpretations for this paradox. Thus, the potential role of IL‐25/IL‐17BR axis in modulating innate immune activation in IPF should be further elucidated.

## 4 IL‐33 IN THE PATHOGENESIS OF IPF: DIFFERENT ISOFORMS DETERMINE THE PRO‐FIBROTIC ROLES

It is well known that IL‐33 acts as a dual‐function protein depending on different cellular localization. Full‐length form of IL‐33 (flIL‐33) is located in cytoplasm or nucleus and acts as transcription factor to promote the expression of target genes, whereas extracellular IL‐33 (i.e., mature IL‐33, mIL‐33) binds with ST2L (membrane‐bound receptors) to activate its cellular targets.^26^ Also, ST2 can shed from cell surface (i.e., sST2) and acts as a “decoy” receptor to bind and block IL‐33's function.^26^

Tajima et al.^27^ showed us that the serum ST2 (sST2) level was significantly increased in patients of IPF with acute exacerbation, which correlated positively with the serum lactate dehydrogenase (LDH) and C‐reactive protein (CRP), whereas inversely correlated with arterial partial pressure of oxygen (PaO_2_), the ratio of PaO_2_ to the fractional concentration of oxygen in inspired gas (PaO_2_/FiO_2_) and the percentage of predicted vital capacity (%VC). This indicated that increased circulating sST2 levels may be the biomarker that reflects the development of the inflammatory milieu in the IPF lung. By using BLM induced pulmonary fibrosis model, the same author also showed that BLM markedly augmented soluble ST2 mRNA expression following with the upregulation of Th2‐type cytokines (IL‐4 and IL‐5) and proinflammatory cytokines (IL‐1β and TNF‐α).^28^ Furthermore, by in vitro cell culture system, they found that the combination of IL‐1β, TNF‐α, and IL‐4 could promote ST2 mRNA expression in both human type II alveolar epithelial cell and lung fibroblast.^28^ Collectively, these findings indicated that elevated sT2 level reflects dys‐regulated local inflammatory response and Th2‐type immune milieu, which may promote the progression of lung fibrosis. However, the pathogenic role of IL‐33 dependent or independent of ST2L axis still remained unclear in these studies.

Luzina et al.^29^ conducted the first study to delineate the potential role of IL‐33 on non‐allergic lung inflammation in both human and animal IPF model. They found that hIL‐33 constitutively expressed in healthy lungs, whereas the expression was much higher in the lungs of IPF patients, with inflammatory cells and fibroblasts in fibrotic foci to be the most intense staining cells. By instillation intratracheally with AdV expressing mice‐flIL‐33 and mice‐mIL‐33, they showed us that flmIL‐33 mainly recruited the infiltration of lymphocytes and neutrophils, whereas profound eosinophilia together with increased lymphocytes and neutrophils were observed in mIL‐33 transfection mice. Furthermore, they found that both mIL‐33 and flIL‐33 can induce a profound inflammatory milieu in lung parenchyma by inducing the production of IL‐1β, TNF‐α, IL‐6, macrophage inflammatory protein (MIP)‐1α, and RANTES. mIL‐33, but not flIL‐33, also promote a skew toward the Th2 cytokine response by up‐regulating the expressions of IL‐4, IL‐5, and IL‐13. Furthermore, deficiency of ST2L remarkably attenuated the mIL‐33‐induced pulmonary eosinophilia and the local expression of IL‐4 and IL‐5. By contrast, ST2 deficiency failed to attenuate pulmonary inflammatory cells infiltration and cytokines production induced by flIL‐33.^29^ Taken together, we concluded from this study that both flmIL‐33 and mmIL‐33 can participate in the non‐atopic pulmonary inflammation in a ST2L independent and dependent manner, respectively. In order to further elucidate the potential role of flmIL‐33 on lung fibrotic lesion formation, Luzina et al.^30^ conducted another study by intratracheal instillation with flmIL‐33‐encoding adenovirus to show that flmIL‐33 alone induced significant lung collagen deposition and showed a synergistic effect on the levels of lung collagen a1 (COL1A1) and COL3A1 mRNA induced by BLM. The profibrotic effect of flmIL‐33 was accompanied with the elevated expression of several mediators, including TGF‐β1, monocyte chemotactic protein (MCP)‐1, MIP‐1α, IL‐6, TNF‐α, and HSP70. Thus, in addition to an indicator of lung inflammation, flIL‐33 exhibits a proinflammatory and profibrotic effect in a ST2L and Th2‐independent fashion. Rather, fIL‐33 acts as an intracellular transcription factor to promote the expressions of several key cytokines.

However, the potential role and mechanism of mIL‐33‐sT2L axis on lung fibrosis still remained undefined in the previously discussed studies. Thus, Li et al.^31^ evaluated the profibrotic effect of mIL‐33 in BLM‐induced lung fibrosis model by using ST2L^−/−^C57BL/6 mice. They showed that BLM‐induced inflammatory and fibrotic changes were significantly reduced in ST2L^−/−^ mice, as manifested by impaired inflammatory cell recruitment, reduced pulmonary cytokines expression, collagen deposition, and histologic inflammatory/fibrosis scores. Likewise, they found that anti‐IL‐33 antibody notably attenuated BLM‐induced lung inflammation and fibrosis, whereas administration of exogenous mIL‐33 markedly potentiated BLM‐induced lung inflammation, collagen deposition, and histologic changes score. More importantly, Li et al. also delineated the potential cellular targets of mIL‐33.^31^ They first showed that alveolar macrophage depletion can remarkably block BLM or BLM plus IL‐33 challenge‐induced lung information and fibrosis. Also, they found that BLM treatment markedly increased the number of lung M2‐macrophages (CD11b^+^F4/80^+^CD206^+^), which can be significantly disrupted by ST2L depletion. This indicated that mIL‐33‐ST2L axis may drive the polarization of macrophages toward M2 in response to BLM. This was confirmed by the in vitro study that showed that mIL‐33 can promote IL‐13 expression and potentiate IL‐13‐induced Arg1 (a biomarker of M2‐macrophage) expression in bone marrow (BM)‐derived macrophages. It was reported that lung macrophages contain two major distinct phenotypes, a pro‐inflammatory subset (M1, i.e., classically activated macrophages) that can generate a variety of pro‐inflammatory cytokine, and an anti‐inflammatory, pro‐fibrotic subset (M2, i.e., alternatively activated macrophages) that can potentiate abnormal tissue repair and wound healing processes.^32^ Thus, we can draw the conclusion that mIL‐33‐ST2L axis may promote lung fibrosis by recruiting M2‐macrophages. Furthermore, Li et al.^31^ reported that BLM dramatically enhanced the lineage^−^ICOS[inducible costimulator]^−^ST2L^+^ILC2 recruitment, which was almost completely blocked by ST2L deletion. Conversely, ILC2 transfer notably exacerbated lung inflammation and fibrosis induced by BLM plus IL‐33, which was accompanied by upregulation of collagen III, IL‐13, and TGF‐β1 in lung tissues. Thus, this study indicates that mIL‐33‐ST2L axis plays a significant role in the initiation and maintenance of lung inflammation and fibrosis in a M2‐macrophages and ILC2s‐dependent fashion. This concept was further confirmed by several other studies. For example, by intranasal instillation of soluble ST2 (sST2, a endogenous decoy receptor of mIL‐33‐ST2L axis), Gao et al.^33^ showed that BLM‐induced expression levels of IL‐4, IL‐6, IL‐13, IL‐33, MCP‐1, and TGF‐β1 were all markedly reduced by intranasal instillation of soluble ST2, which is followed by attenuated pulmonary inflammatory cell infiltration, reduced fibrotic lesions, and improved outcomes. Fanny et al.^34^ in their recently published study also found that ST2L‐deficiency significantly blocked BLM‐induced lung recruitment of CD11b^+^F4/80^+^CD206^+^ alternative (M2) macrophages, suggesting that IL‐33/ST2L signaling pathway mediates BLM‐induced M2 skew in vivo. Finally, Zhao et al.^35^ found that ILC2 recruitment, IL‐13 expression, collagen deposition, and fibrosis degree were all significantly counteracted by ST2L‐knockout in BM, indicating that IL‐33/ST2L axis promotes lung injury and fibrosis by recruiting of BM‐derived ILC2s.

Taken together, we conclude that both flIL‐33 and mIL‐33 are important profibrotic mediators by distinct mechanisms. Briefly, flIL‐33 exerts its proinflammatory and profibrotic effects in a ST2L and Th2‐independent fashion, but via modulating gene expressions of several non‐Th2 cytokine and HSP70. By contrast, mIL‐33 may potentiate lung fibrosis by recruiting ST2L^+^M2‐macrophages and ST2L^+^ILC2s to enlarge Th2 cytokine milieu by excessive production of IL‐4, IL‐5, and IL‐13.

## 5 TSLP/TSLPR AXIS IN THE PATHOGENESIS OF IPF: DIRECTLY ACTING ON FIBROBLAST

TSLP was first implicated as an initiator of Th2 immune response in the airways.^36^ Previous studies showed that aberrant levels of TSLP contributed to the pathogenesis of allergic inflammatory conditions (including allergic asthma, nasal polyps, and atopic dermatitis) by promoting the polarization of immature DCs into a type 2 phenotype, and driving excessive type 2 cytokine responses.^37^ TSLP exerts its biological effects by binding to a functional heterodimeric receptor complex, which is composed of the TSLP receptor (TSLPR) and the IL‐7Rα‐chain, and activating STAT3/STAT5 signaling pathways.^37^ Recently, TSLP has also been shown as an important cytokine in the pathogenesis of non‐atopic pathobiology dominated by unreasonable type 2 cytokine responses, including both cutaneous and lung fibrotic diseases.^38^, ^39^ Datta et al.^40^ using a small cohort of IPF patients (*N* = 12) reported that TSLP/TSLPR axis was remarkably upregulated in patients with IPF. They found that fibrotic lung tissues from patients with IPF showed obvious TSLP immunoreactivity predominantly in alveolar epithelial cells (AECs) and myofibroblasts within fibrotic foci, indicating both AECs and mesenchymal cells are the potential cellular sources of TSLP. They also showed that TSLPR immunostaining was positive on airway smooth muscle cells (ASMC), AECs, and myofibroblasts, indicating that myofibroblasts act as both cellular resources and cellular targets of TSLP in patients with IPF. In accordance with this study, Lee et al.^25^ showed that BALF level of was significantly higher in the IPF group than that in normal control subjects, indicating TSLP can be served as an disease marker for IPF. Datta et al.^40^ also conducted the in vitro study to examine the potential pathogenic role of TSLP on lung fibrosis. They demonstrated that primary human lung fibroblasts (pHLFs) expressed a functional TSLP receptor complex, that is, TSLPR and IL‐7Ra. Furthermore, exogenous administration of TSLP can significantly increases CCL2 mRNA and protein level produced by pHLFs, suggesting that human pHLFs constitutively express TSLPR and can respond to TSLP by increasing chemokine expression. Taken together, we hypothesized that in addition to modulate allergic/atopic inflammation, TSLP may also play a key role in the pathogenesis of non‐allergic fibrotic diseases dominated by a type 2 phenotype skew. The potential mechanism underlying the profibrotic effect may be that TSLP promotes TSLPR^+^ fibroblasts to form excessive cytokines/chemokines milieu, which eventually leads to dysregulated remodeling process and scar formation.

## 6 CONCLUSIONS AND FUTURE DIRECTIONS

**FIGURE 2:**
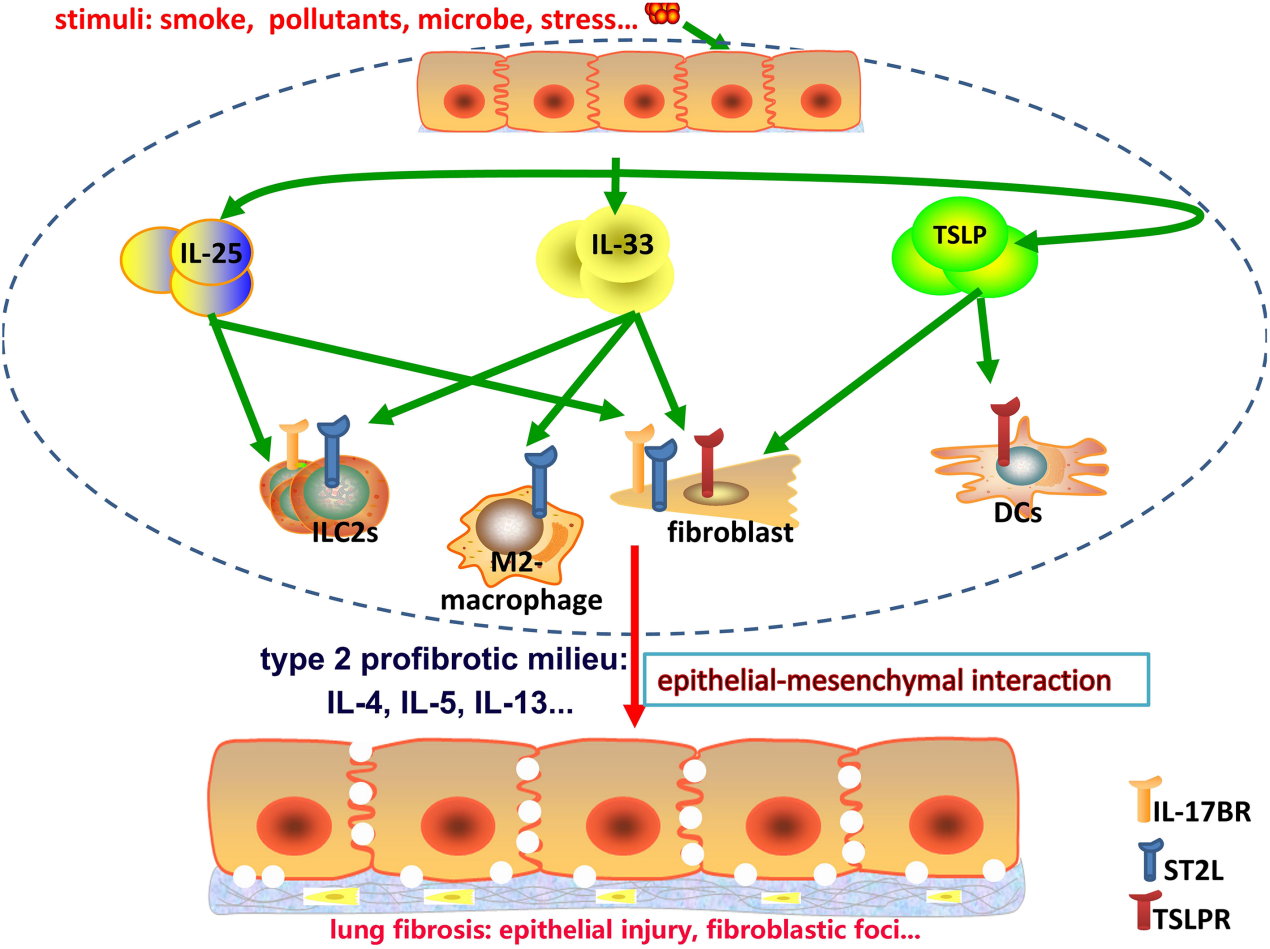
Schema diagram for the role of IL‐25/IL‐33/TSLP on the pathogenesis of IPF. IL‐25/IL‐33/TSLP mainly derived from epithelial cells in response to cigarette smoke, microbe, pollutant, and stress. Increased alveolar levels of IL‐25/IL‐33/TSLP form a cytokine milieu that can recruit a variety of target cells, which express their corresponding receptors (i.e., IL‐17BR/ST2L/TSLPR), including type 2 innate lymphoid cells (ILC2s), M2‐macrophages, dendritic cells (DCs), and fibroblasts. Thus, dys‐regulated crosstalk between epithelial cells and mesenchymal cells (including resident structure cells and infiltrating innate immune cells) forms and initiates the skew of type 2 cytokine response by excessive production of IL‐4, IL‐5, and IL‐13. This uncontrolled type 2 immunity promotes continued activation of fibroblasts and epithelial injury, which will ultimately lead to excessive collagen production and irreversible fibrotic lesions. Thus, early and combined targeting of these cytokines may block the progressive type 2‐initiated fibrotic disease. Abbreviations: IL, interleukin; IPF, idiopathic pulmonary fibrosis; TSLP, thymic stromal lymphopoietin

Herein, we hypothesize that in addition to mediate allergic airway inflammation and remodeling, epithelium‐derived triple cytokines, IL‐25/IL‐33/TSLP, can also potentiate non‐atopic lung parenchymal inflammation and fibrogenesis mainly by initiating and propagating type 2 immunity. We also suggest that IL‐25/IL‐33/TSLP axis may be the master regulator of abnormal epithelial–mesenchymal interaction and serve as a novel treatment target for chronic fibrotic lung disease, especially IPF (Figure 2). However, these concepts must be further tested in future studies. First, large IPF cohort of prospective or retrospective clinical studies on IL‐25/IL‐33/TSLP is needed. The important roles and mechanisms of IL‐25/IL‐33/TSLP are mainly elucidated by experimental murine lung fibrosis model induced by BLM or *S. mansoni egg*s. Unfortunately, until now there is no murine model can recapitulate all the features of usual interstitial pneumonia (UIP),^41^, ^42^ which is the pathological hallmarks of IPF. Thus, there may be unexpected traps when interpreting these experimental findings into clinical practice. Although previous studies also showed that IL‐25/IL‐33/TSLP was up‐regulated in alveolar milieu of IPF patients,^16^, ^22^, ^29^, ^30^ the clinical implications still remain poorly defined because of the small number of enrolled subjects. Unexpectedly, IL‐25 levels in BALF of 100 patients with IPF were not significantly different from normal control subject in a recent relatively large cohort study.^25^ Thus, multicenter, large cohort studies on the exact interactions between IL‐25/IL‐33/TSLP and different IPF phenotypes (e.g., mild or severe, stable state, or acute exacerbation) should be further conducted. Second, murine studies should further elucidate the potential mechanisms of IL‐25/IL‐33/TSLP on lung inflammation and fibrosis. The cellular targets of IL‐25/IL‐33/TSLP vary from innate/adaptive immune cells to lung structural cells, whereas the master target cells that mediate pro‐inflammatory and pro‐fibrotic effect of IL‐25/IL‐33/TSLP on IPF are still undermined. Third, considering the redundant nature of cytokines, future studies should focus on whether combined targeting of IL‐25/IL‐33/TSLP is necessary to completely block the progressive fibrotic lung diseases. In a chronic HDM‐induced allergy and *Schistosome egg‐*induced lung granuloma model, Vannella et al. showed that early combined blockade of IL‐25/IL‐33/TSLP by monoclonal antibody (mAb) treatment can suppresses type 2 cytokine‐driven lung inflammation and fibrosis. However, these synergistic effects should be further assessed in other fibrotic murine models.^43^

In conclusion, IL‐25/IL‐33/TSLP may be the master switch that drives the abnormal epithelial–mesenchymal crosstalk in IPF according to preclinical studies. However, until now there are no clinical trials that are under way to evaluate the therapeutic potential of anti‐IL‐25/IL‐33/TSLP strategies. We strongly suggest to use singly or in combination with anti‐IL‐25/IL‐33/TSLP agents in the future clinical trials in order to further improve the clinical outcomes for IPF patients.

## CONFLICT OF INTEREST

The authors declare that there are no competing interests.

## ETHICS STATEMENT

None.

## AUTHOR CONTRIBUTIONS

Z. J. and D. H. conceive the idea; X. X. searched literatures and wrote the manuscript; D. H. critically revised the manuscript. All the authors approved the final version for the publication.

## Data Availability

The data supporting the ideas proposed in this article are available within the article.
